# Supplementary material for: Colossal topological Hall effect at the transition between isolated and lattice-phase interfacial skyrmions
Source: Nat Commun. 2021 May 12;12:2758. doi: 10.1038/s41467-021-22976-6 (PMC8115237; doi:10.1038/s41467-021-22976-6)
Supplement: Supplementary file 1 — Supplementary Information [file 41467_2021_22976_MOESM1_ESM.pdf]

Supplementary Information for
Colossal topological Hall effect at the transition between isolated and lattice-phase interfacial skyrmions

M. Raju[1†‡], A.P. Petrović[1], A. Yagil[2], K.S. Denisov[3], N.K. Duong[1], B. Göbel[4], E. Şaşıoğlu[4], O.M. Auslaender[2⋆], I. Mertig[4], I.V. Rozhansky[3], C. Panagopoulos[1†]

[1]Division of Physics and Applied Physics, School of Physical and Mathematical Sciences, Nanyang Technological University, 637371 Singapore

[2]Department of Physics, Technion, Haifa 32000, Israel

[3]Ioffe Institute, Politekhnicheskaya 26, 194021 St.Petersburg, Russia

[4]Institut für Physik, Martin-Luther-Universität Halle-Wittenberg, 06099 Halle (Saale), Germany

[‡]Present address: Institute for Quantum Matter and Department of Physics and Astronomy, Johns Hopkins University, Baltimore, MD, USA

[⋆]Present address: Neuroscience Institute and Tech4Health Institute, NYU Langone Health, New York, NY

[†]Correspondence: mraju@ntu.edu.sg/christos@ntu.edu.sg

Contents

1 Evolution of magnetic textures **2**

1.1 Evolution of isolated skyrmions and skyrmion lattices with applied field 2

1.2 Evolution of magnetic textures across $T-\kappa$ parameter space 5

1.3 Quantitative analysis of skyrmion configurations 6

2 Magnetic parameters and estimation of $\kappa(T)$ **12**

3 Temperature dependent $\rho^{THE}(H)$ profiles **14**

3.1 $\rho^{THE}(H)$ profiles with Fe/Co composition and the number of repeats in a stack 15

4 Correlation between the enhancement in ρ^{THE}, skyrmion configuration transition and sign reversal of R_0 **16**

5 Insights into the Hall effect in a multi-band system **17**

5.1 Semiclassical argumentation 17

5.2 Explicit transport calculation using Berry curvature 18

6 Density functional theory calculations **21**

1 Evolution of magnetic textures

1.1 Evolution of isolated skyrmions and skyrmion lattices with applied field

Figures S1-S3 show the evolution of isolated and dense skyrmion configurations with applied magnetic field (H) for the samples discussed in figure 1 of the main paper. These multilayers show labyrinthine stripe domains at $H = 0$. For $\kappa < 1$ textures evolve with H as: stripe domains to isolated skyrmions and then to a uniform polarized phase. For $\kappa \geq 1$ textures evolve as: stripe domains to a skyrmion lattice, then to isolated skyrmions and finally to a uniform polarized phase.

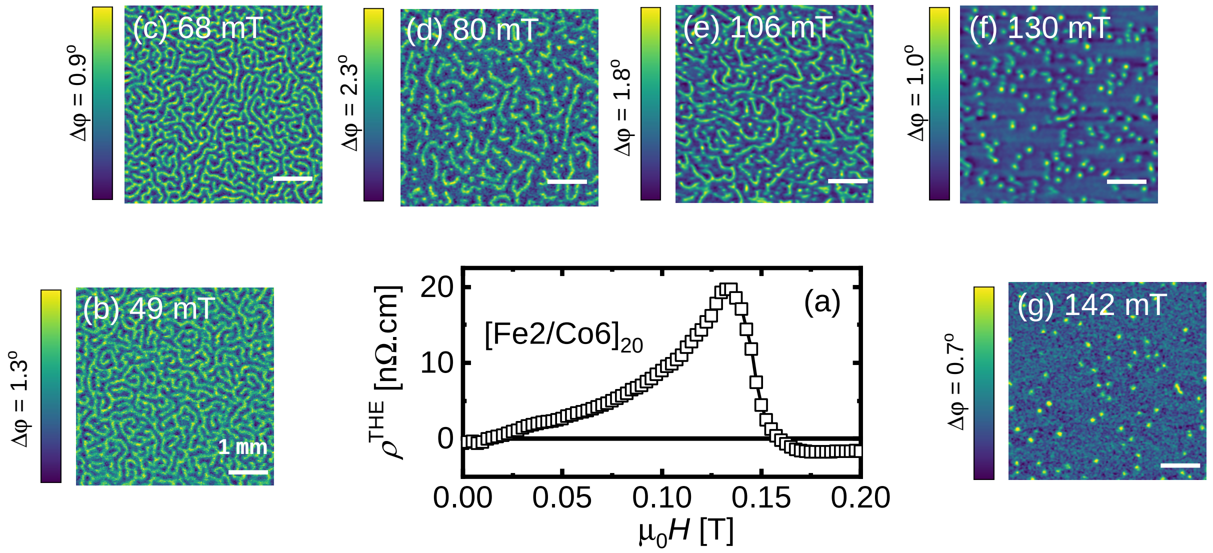

Figure S1: **Evolution of magnetic textures and topological Hall resistivity ($\rho^{THE}(H)$) with applied field in Si/SiO$_2$/[Ir**10**/Fe**2**/Co**6**/Pt**10**]$_{20}$: (a)** Evolution of $\rho^{THE}(H)$. **(b)-(g)** MFM images are acquired at the field values indicated on each image. Scale bar for MFM images is 1 μm, the color scales represents the phase shift in MFM signal due to the magnetic force acting on the tip. Images are acquired at a scan height of 20 nm.

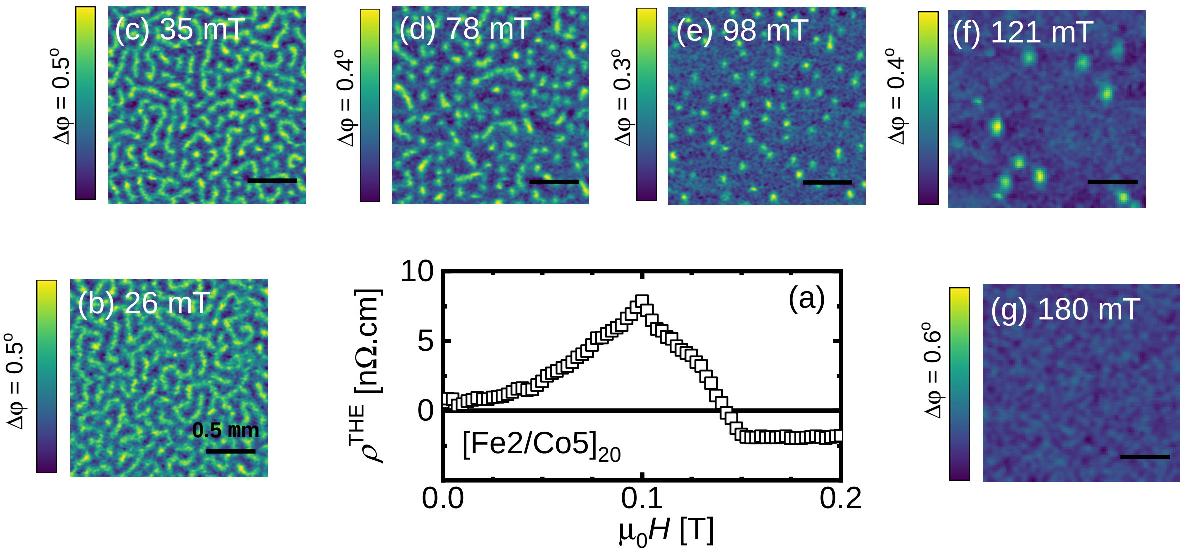

Figure S2: **Evolution of magnetic textures and topological Hall resistivity ($\rho^{THE}(H)$) with applied field in Si/SiO$_2$/[Ir10/Fe2/Co5/Pt10]$_{20}$: (a)** Evolution of $\rho^{THE}(H)$. **(b)-(g)** MFM images are acquired at the field values indicated on each image. Scale bar for MFM images is 0.5 μm, the color scales represents the phase shift in MFM signal due to the magnetic force acting on the tip. Images are acquired at a scan height of 20 nm.

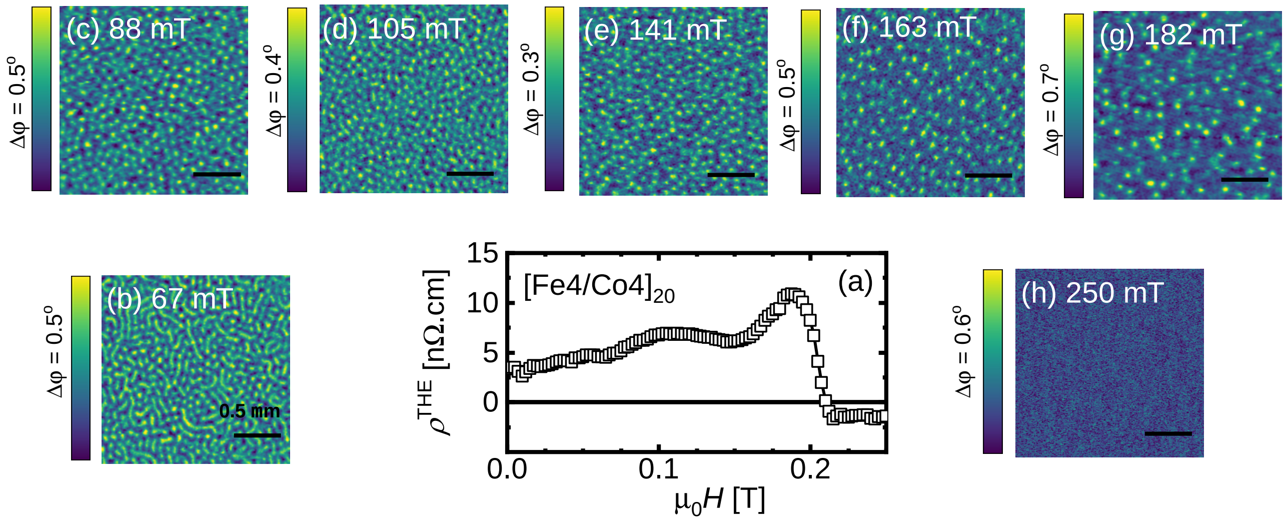

Figure S3: **Evolution of magnetic textures and topological Hall resistivity ($\rho^{THE}(H)$) with applied field in Si/SiO$_2$/[Ir10/Fe4/Co4/Pt10]$_{20}$: (a)** Evolution of $\rho^{THE}(H)$. **(b)-(h)** MFM images are acquired at the field values indicated on each image. Scale bar for MFM images is 0.5 μm, the color scales represents the phase shift in MFM signal due to the magnetic force acting on the tip. Images are acquired at a scan height of 20 nm.

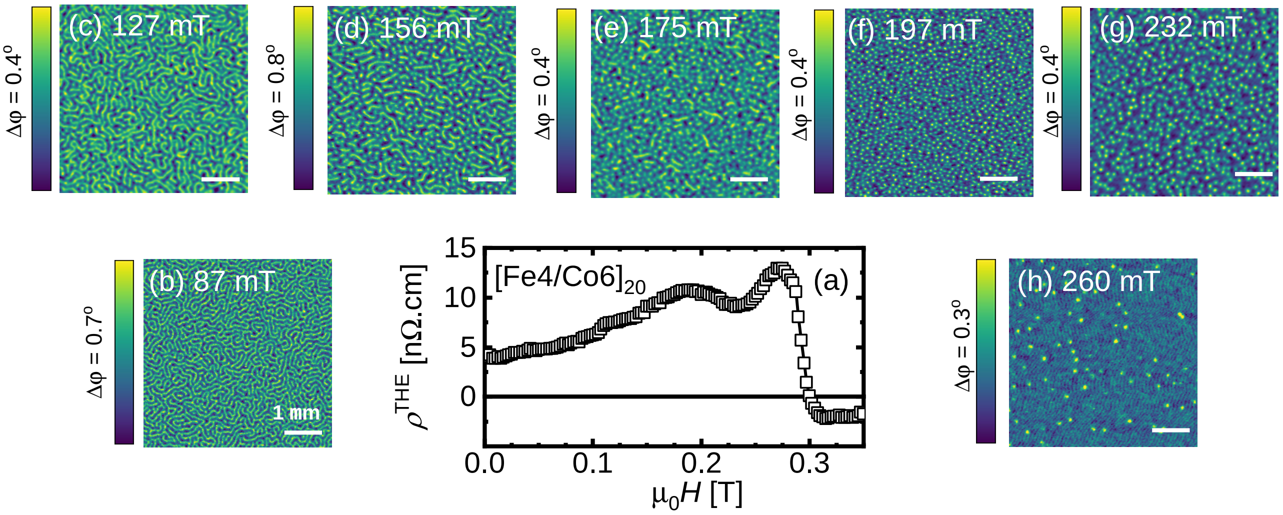

Figure S4: **Evolution of magnetic textures and topological Hall resistivity ($\rho^{THE}(H)$) with applied field in Si/SiO$_2$/[Ir10/Fe4/Co6/Pt10]$_{20}$: (a)** Evolution of $\rho^{THE}(H)$. **(b)-(h)** MFM images are acquired at the field values indicated on each image. Scale bar for MFM images is 1 μm, the color scales represents the phase shift in MFM signal due to the magnetic force acting on the tip. Images are acquired at a scan height of 20 nm.

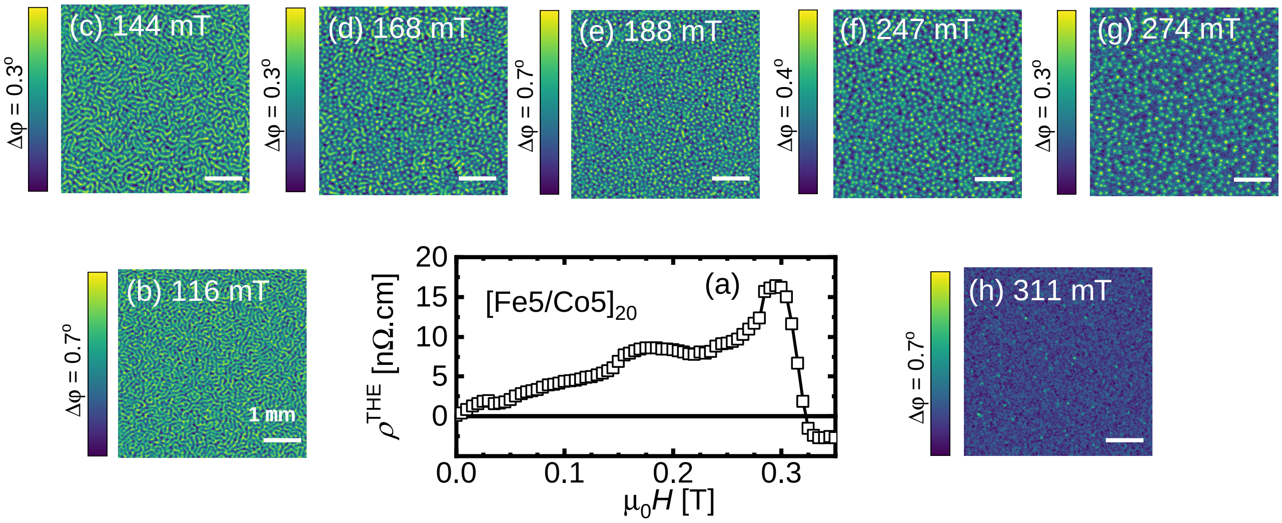

Figure S5: **Evolution of magnetic textures and topological Hall resistivity ($\rho^{THE}(H)$) with applied field in Si/SiO$_2$/[Ir10/Fe5/Co5/Pt10]$_{20}$: (a)** Evolution of $\rho^{THE}(H)$. **(b)-(h)** MFM images are acquired at the field values indicated on each image. Scale bar for MFM images is 1 μm, the color scales represents the phase shift in MFM signal due to the magnetic force acting on the tip. Images are acquired at a scan height of 20 nm.

1.2 Evolution of magnetic textures across $T - \kappa$ parameter space

Figure S6 shows the evolution of the magnetic textures across the T and κ parameter space discussed in figure 2a of the main paper.

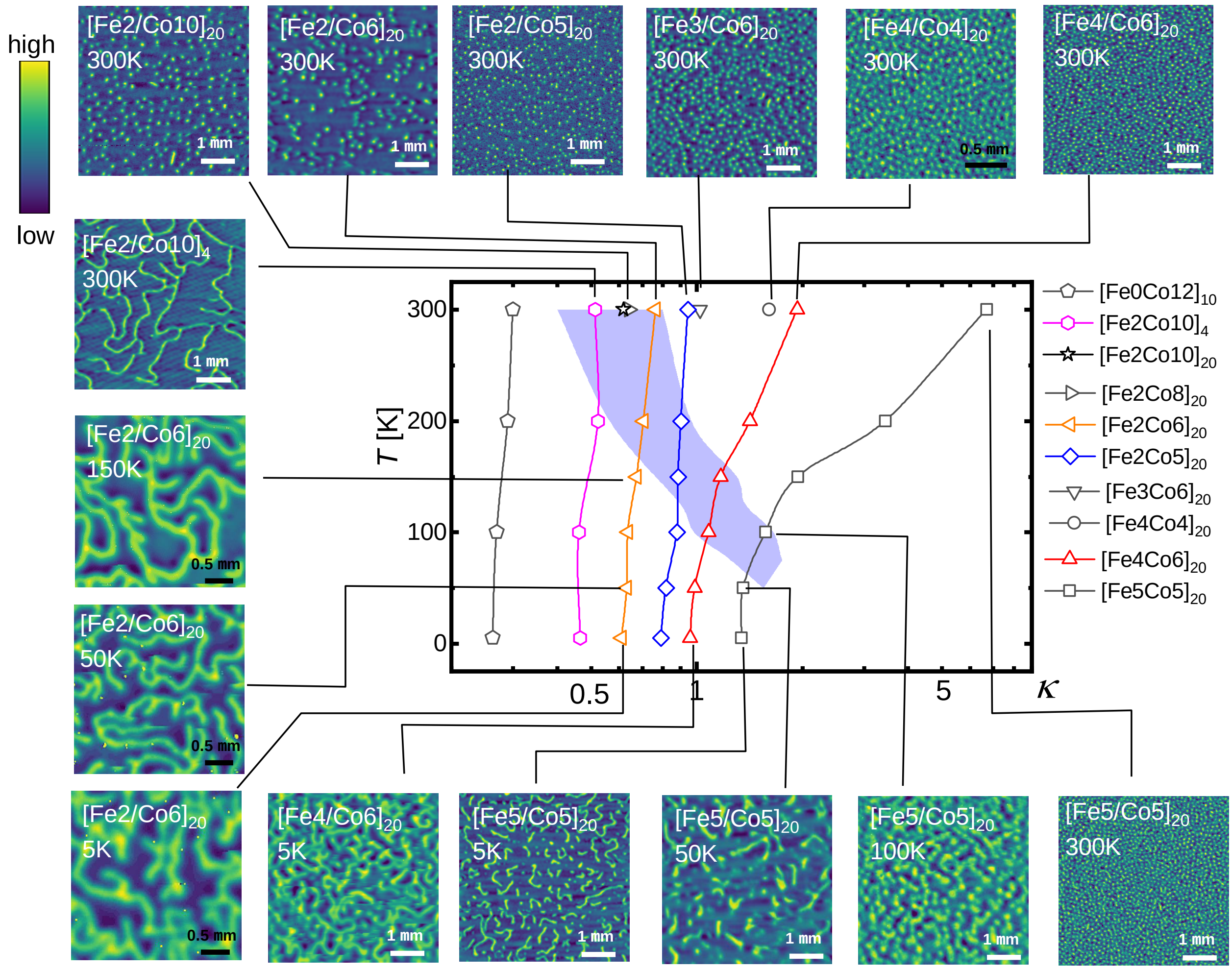

Figure S6: **Evolution of magnetic textures across $T - \kappa$ parameter space.** The color scales represents the change in MFM signal due to the magnetic force acting on the tip. The scale bar for each image is indicated in the image itself as the images were of different sizes.

1.3 Quantitative analysis of skyrmion configurations

Figure S7-S11 shows the analysis of spatial configuration of skyrmions for varying Fe/Co composition and H presented in Fig.S1-S5. The images are analysed using Delaunay triangulation for nearest neighbour (NN) coordination number (N_{NN}) and their angular orientation (θ_{NN}). Dense skyrmion arrays show a configuration with dominant population having $N_{NN} = 6$ and θ_{NN} close to 60°. These statistics indicate an evolution of a disordered hexagonal lattice as the skyrmion density increases.

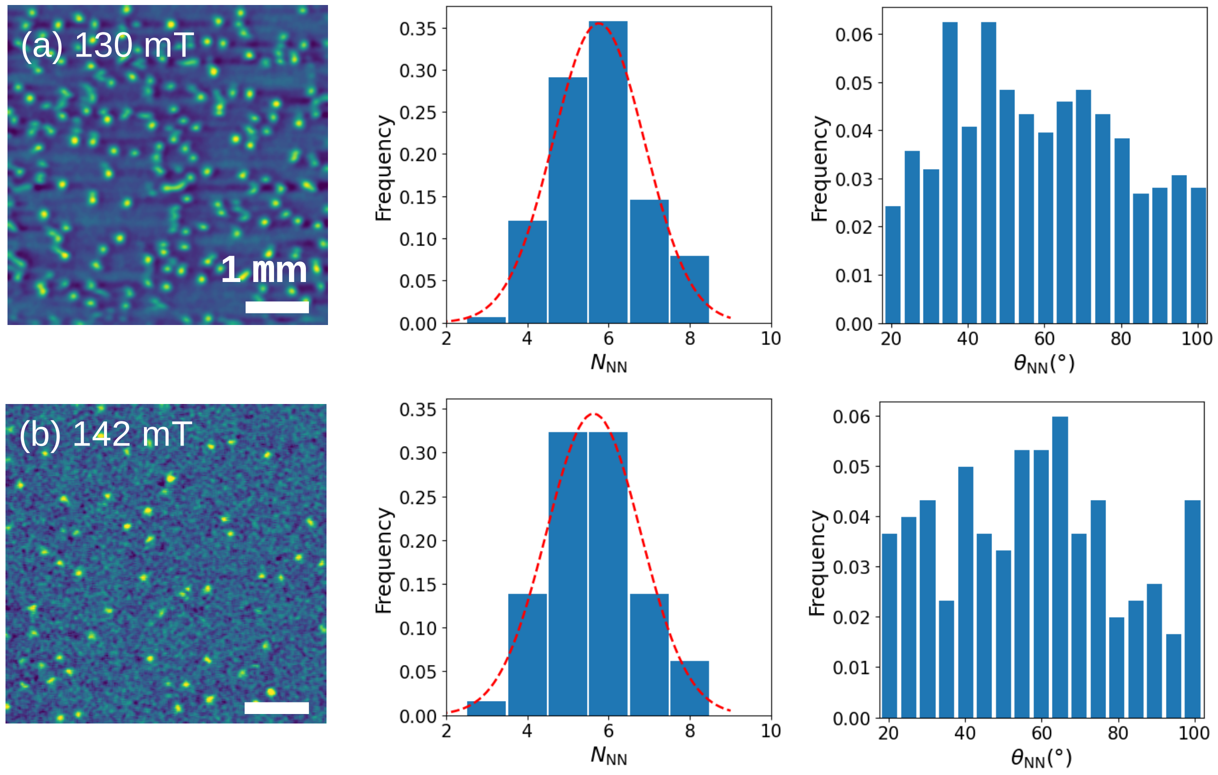

Figure S7: **Quantitative analysis of skyrmion configurations in Si/SiO$_2$/[Ir10/Fe2/Co6/Pt10]$_{20}$: (a)** Isolated skyrmion configuration observed at $H = 130$ mT. Nearest neighbour (NN) statistics: NN coordination number (N_{NN}) and their angular orientation (θ_{NN}). **(b)** Isolated skyrmion configuration observed at $H = 142$ mT and the corresponding N_{NN} and θ_{NN}. MFM images are reproduced from figure S1

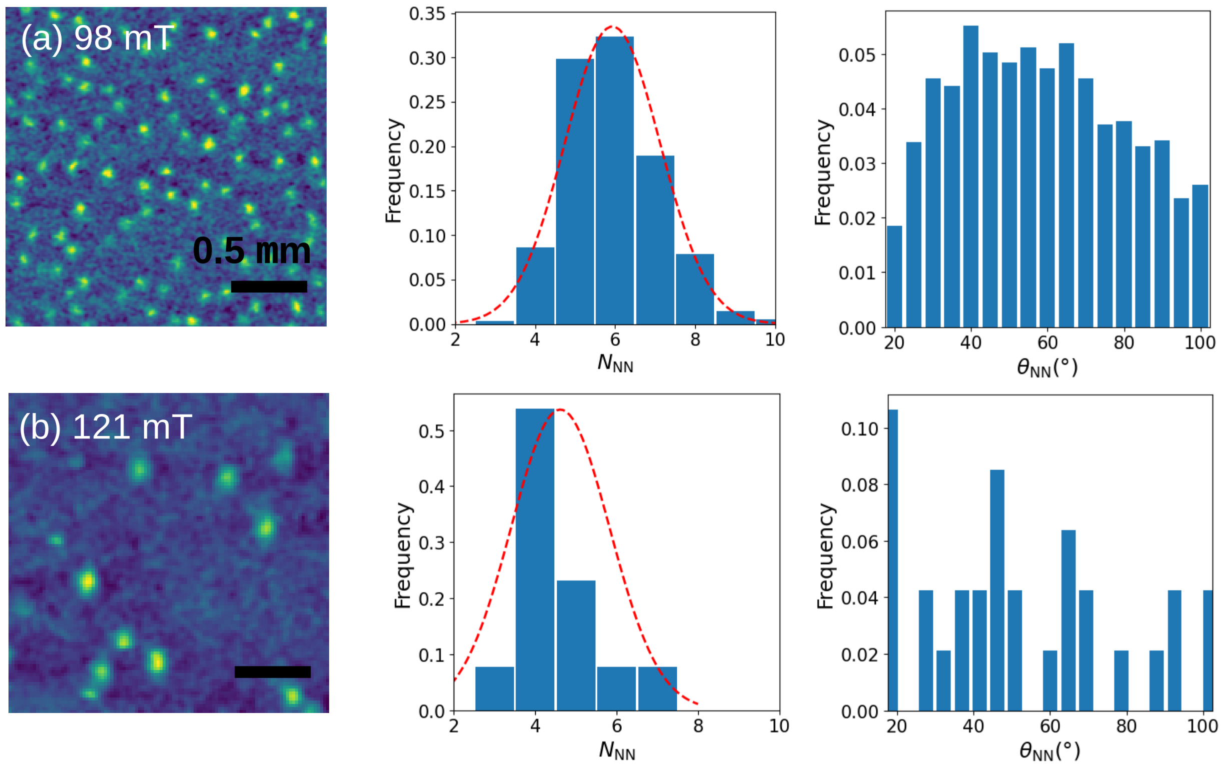

Figure S8: **Quantitative analysis of skyrmion configurations in Si/SiO$_2$/[Ir10/Fe2/Co5/Pt10]$_{20}$: (a)** Isolated skyrmion configuration observed at $H = 98$ mT. Nearest neighbour (NN) statistics: NN coordination number (N_{NN}) and their angular orientation (θ_{NN}). **(b)** Isolated skyrmion configuration observed at $H = 121$ mT and the corresponding N_{NN} and θ_{NN}. MFM images are reproduced from figure S2

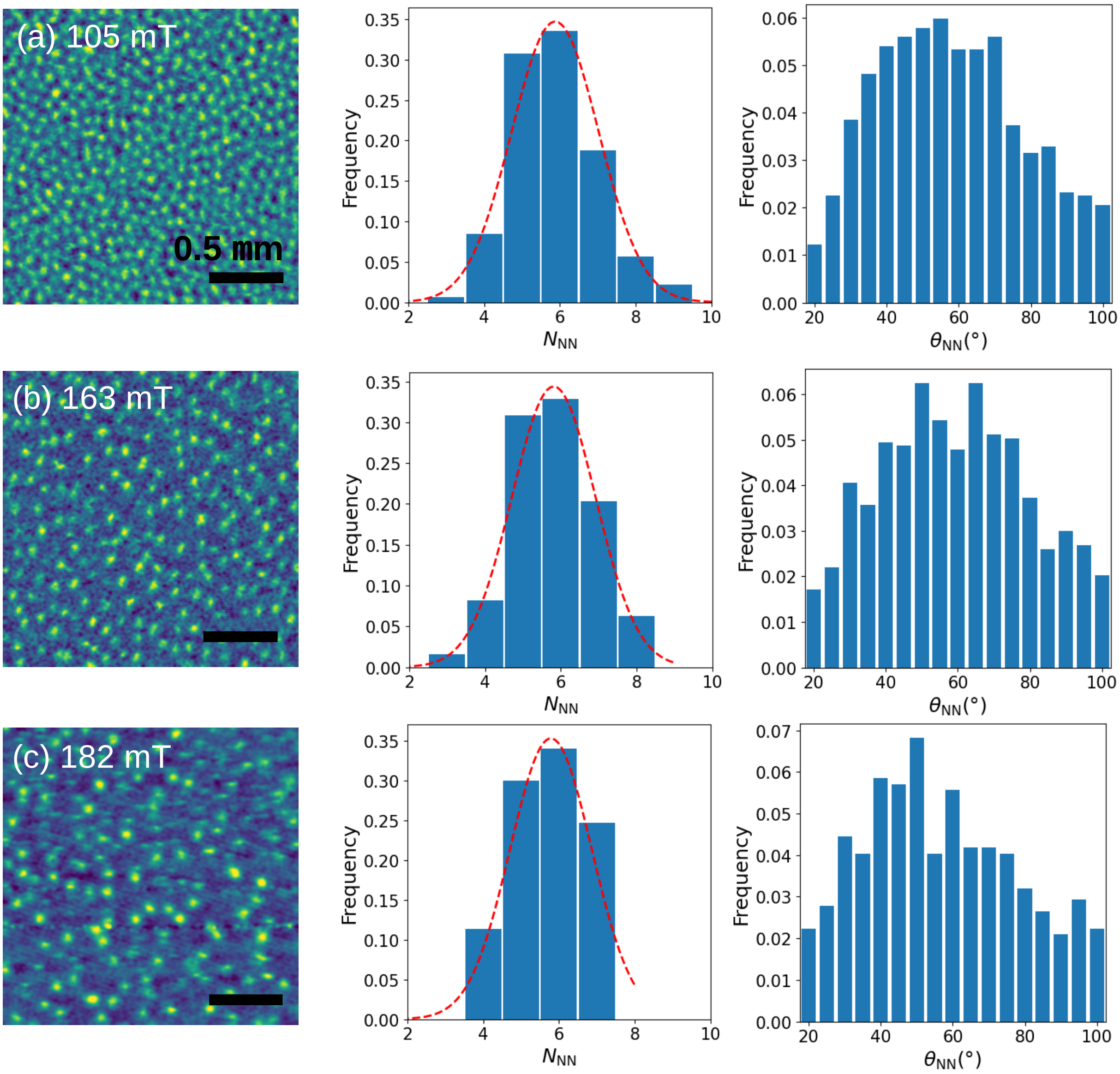

Figure S9: **Quantitative analysis of skyrmion configurations in Si/SiO$_2$/[Ir10/Fe4/Co4/Pt10]$_{20}$: (a)** Dense arrays of skyrmion configuration observed at H = 105 mT. Nearest neighbour (NN) statistics: NN coordination number (N_{NN}) and their angular orientation (θ_{NN}). **(b)** Dense arrays of skyrmion configuration observed at H = 163 mT and the corresponding N_{NN} and θ_{NN}. **(c)** Isolated skyrmion configuration observed at H = 182 mT and the corresponding N_{NN} and θ_{NN}. MFM images are reproduced from figure S3.

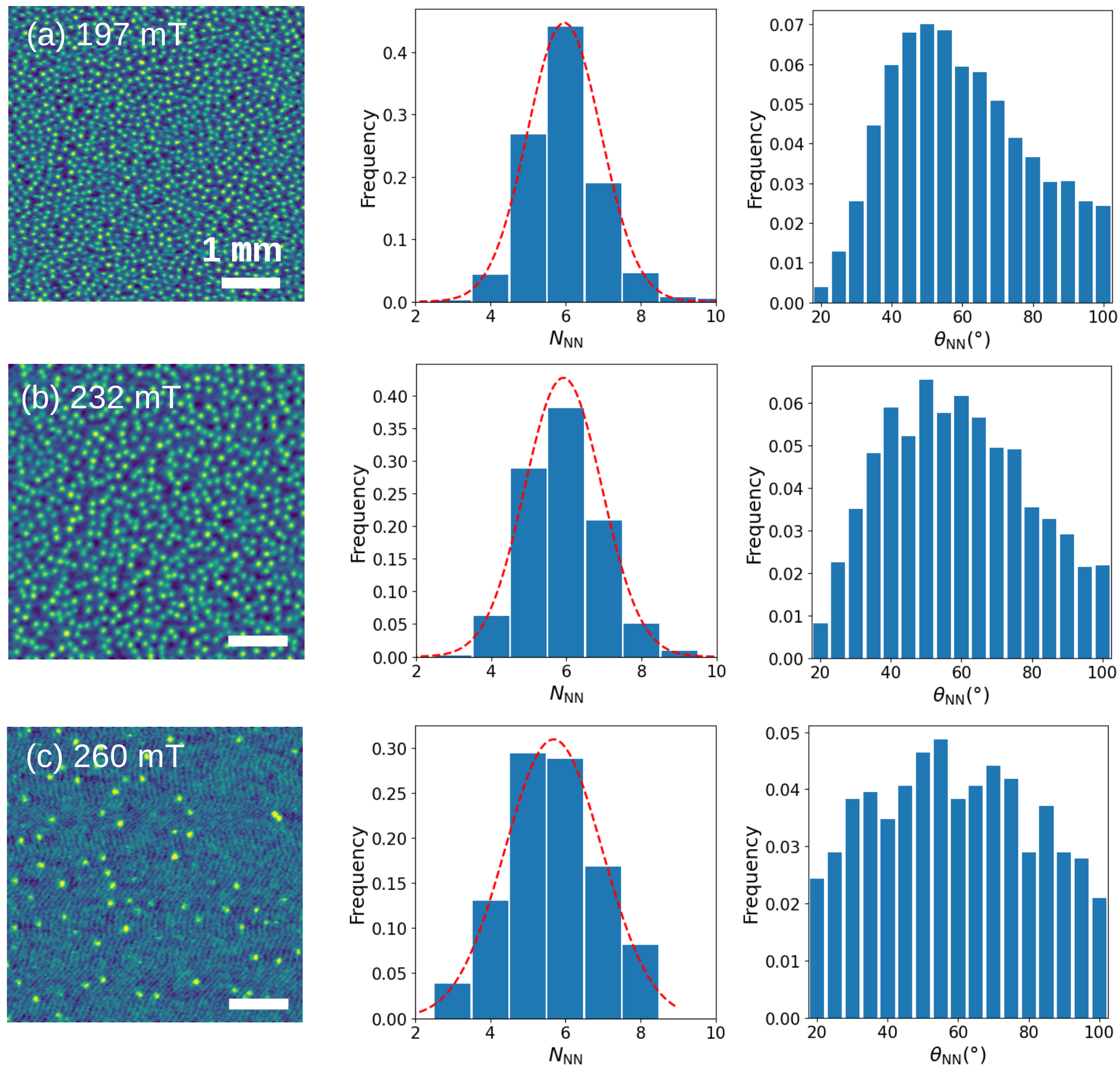

Figure S10: **Quantitative analysis of skyrmion configurations in Si/SiO$_2$/[Ir10/Fe4/Co6/Pt10]$_{20}$: (a)** Dense arrays of skyrmion configuration observed at $H = 197$ mT. Nearest neighbour (NN) statistics: NN coordination number (N_{NN}) and their angular orientation (θ_{NN}). **(b)** Dense arrays of skyrmion configuration observed at $H = 232$ mT and the corresponding N_{NN} and θ_{NN}. **(c)** Isolated skyrmion configuration observed at $H = 260$ mT and the corresponding N_{NN} and θ_{NN}. MFM images are reproduced from figure S4.

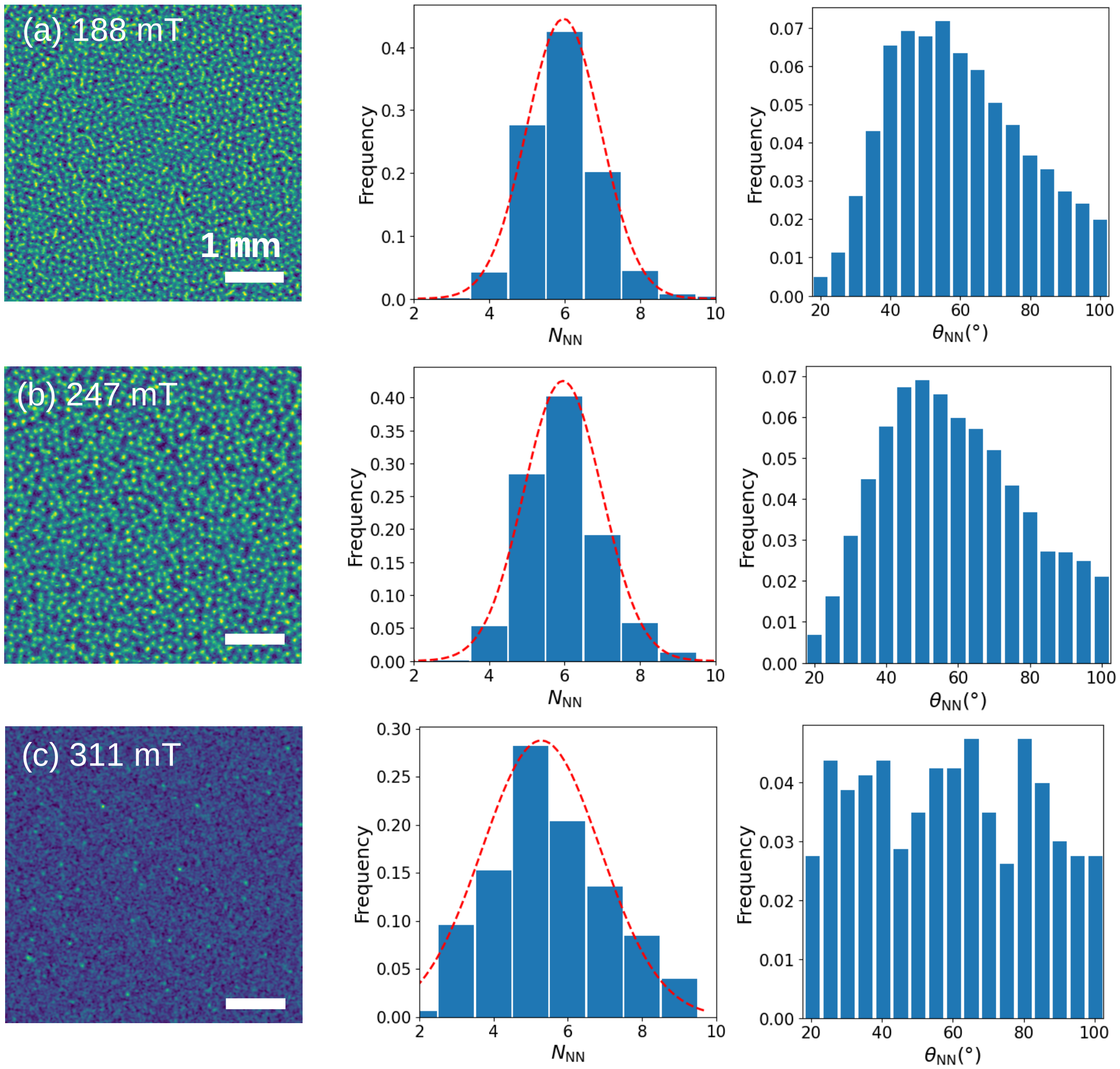

Figure S11: **Quantitative analysis of skyrmion configurations in Si/SiO$_2$/[Ir10/Fe5/Co5/Pt10]$_{20}$: (a)** Dense arrays of skyrmion configuration observed at $H = 188$ mT. Nearest neighbour (NN) statistics: NN coordination number (N_{NN}) and their angular orientation (θ_{NN}). **(b)** Dense arrays of skyrmion configuration observed at $H = 247$ mT and the corresponding N_{NN} and θ_{NN}. **(c)** Isolated skyrmion configuration observed at $H = 311$ mT and the corresponding N_{NN} and θ_{NN}. MFM images are reproduced from figure S5.

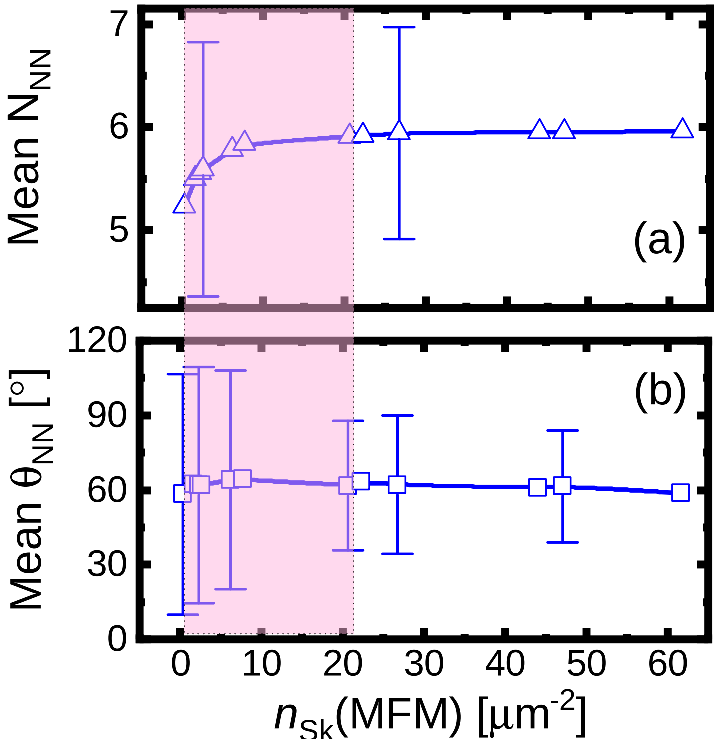

Figure S12: Evolution of the mean value of skyrmion nearest neighbor (NN) **(a)** coordination number (N_{NN}) and **(b)** angular orientation (θ_{NN}) with skyrmion density. The shaded region marks the gradual transition from an isolated skyrmion configuration to a disordered lattice (N_{NN} approaches $\approx$ 6 with a decreasing spread in θ_{NN}). Beyond $n_{Sk}(MFM) = 20$ μm^{-2}, a disordered skyrmion lattice configuration is stabilized. The error bars in each panel indicate the spread (standard deviation) in N_{NN} and θ_{NN}.

To identify the transition from isolated skyrmions to disordered lattice configurations, we analyzed a set of images in figure S12 where we track the variation of the mean value of N_{NN} and θ_{NN} as a function of the MFM-imaged skyrmion density ($n_{Sk}(MFM)$). As shown in Fig. S12(a), the mean value of N_{NN} smoothly approaches a value of $\approx$ 6 for $n_{Sk}(MFM) > 20$ μm^{-2} and the spread (standard deviation) in the values of θ_{NN} (centered around $\approx$ 60°) drops significantly from 48° to 20°, suggesting the development of a disordered lattice skyrmion configuration. These results are consistent with the rapid increase in the estimated discrepancy between imaging and transport for $n_{Sk}(MFM)$ below $20 - 30$ μm^{-2}, as shown in figure 1 of the main paper.

2 Magnetic parameters and estimation of $\kappa(T)$

The magnetic interactions responsible for skyrmion formation are the exchange interaction, quantified by the exchange stiffness (A), the Dzyaloshinskii–Moriya interaction (DMI, D) and the magnetic anisotropy (K_{eff}). Quantification of A and D relies on a method where extensive micromagnetic simulations are employed to reproduce the magnetic texture (zero-field domain structure and field evolution of skyrmion size (d_{sk})) observed in MFM imaging [1, 2, 3], whereas K_{eff} can be determined from the magnetization measurements. The thermodynamic stability parameter ($\kappa \equiv \pi D/4\sqrt{AK_{eff}}$) captures the competition between these magnetic interactions and controls skyrmion properties, such as density (n_{sk}) and d_{sk}. For $\kappa < 1$ skyrmions exist as isolated entities with a larger d_{sk} compared to $\kappa \geq 1$ where skyrmions form dense arrays with a reduced d_{sk}. The details on room temperature (RT) magnetic interactions and their quantification for the samples studied in this work are shown in our earlier work [3, 4, 5]. In the current work we use a T dependent saturation magnetization ($M_S(T)$) to estimate $A(T)$ and $D(T)$ across various samples for $T = 5 - 300$ K.

Figure S13 shows the $M_S(T)$ measured from SQUID and the estimated K_{eff} from $M(H)$ data recorded in out-of-plane and in-plane applied field. $M_S(T)$ is then used together with previously quantified A and D [3] to estimate $A(T)$ and $D(T)$ using the scaling relations [6] shown in eqn. 1

$$\frac{A(T)}{A(T = 5\ K)} = \left[\frac{M_S(T)}{M_S(T = 5\ K)}\right]^{1.5}, \qquad \frac{D(T)}{D(T = 5\ K)} = \left[\frac{M_S(T)}{M_S(T = 5\ K)}\right]^{1.5}. \tag{1}$$

We highlight that variation of $T = 5 - 300\ K$ results in a larger change in $K_{eff}(T)$ compared to $A(T)$ and $D(T)$. As a result, $\frac{1}{\sqrt{K_{eff}}}$ plays the dominant role compared to $\frac{D}{\sqrt{A}}$ in the estimated $\kappa(T) = \frac{\pi D(T)}{4\sqrt{A(T)*K_{eff}(T)}}$. As shown in figure S14, depending on sample composition, $\frac{D}{\sqrt{A}}$ changes only by $5 - 25\%$ while $\frac{1}{\sqrt{K_{eff}}}$ changes by $25 - 500\%$. This shows that the estimated $\kappa(T)$ is more sensitive to the experimentally determined quantity $K_{eff}(T)$, rather than the estimated $A(T)$ and $D(T)$.

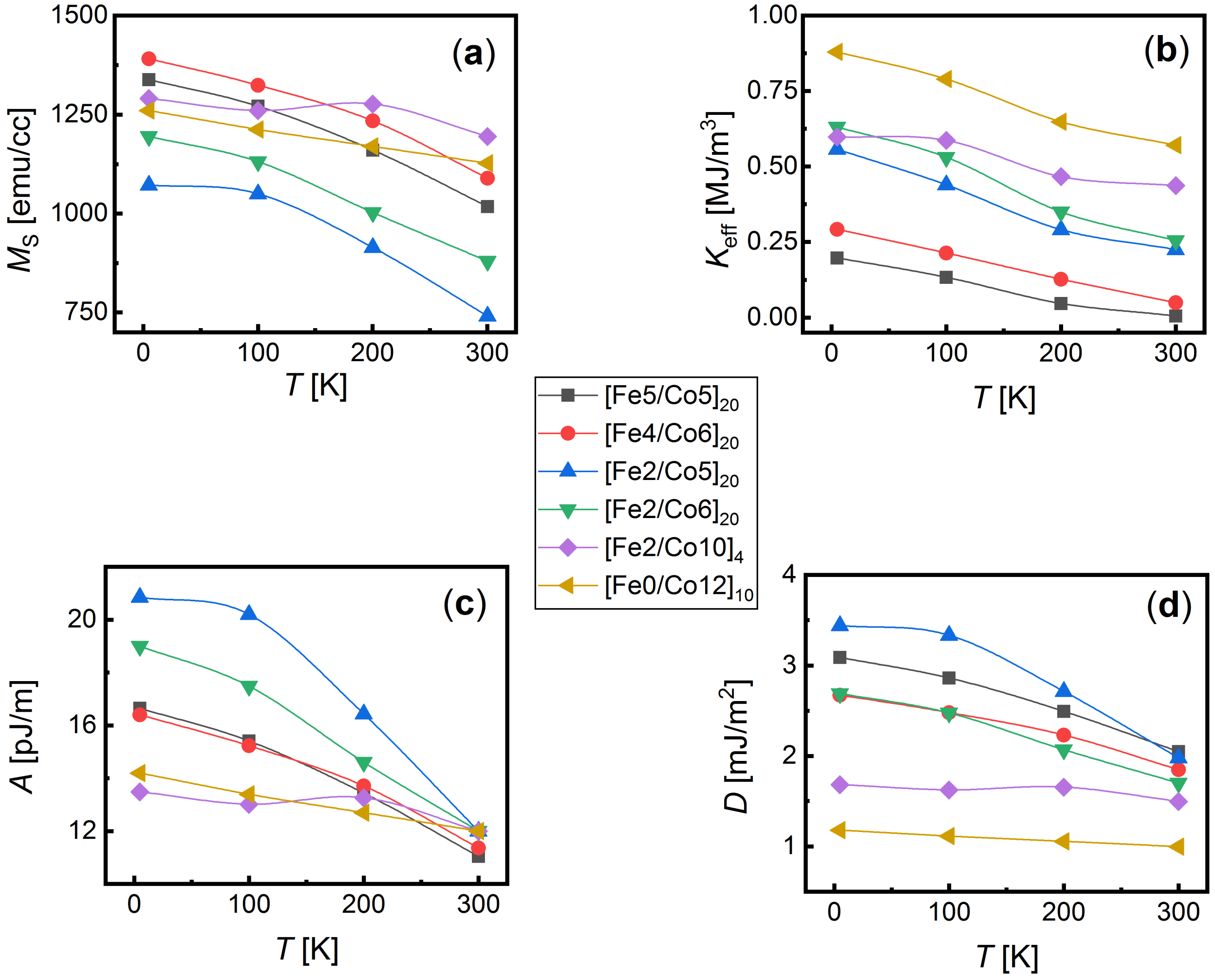

Figure S13: **Temperature variation of M_S, K_{eff}, A and D: (a)** Saturation magnetization measured with a SQUID magnetometer. **(b)** K_{eff} estimated from $M(H)$ data. **(c)** Estimated $A(T)$ using eqn. 1. **(d)** Estimated $D(T)$ using eqn. 1.

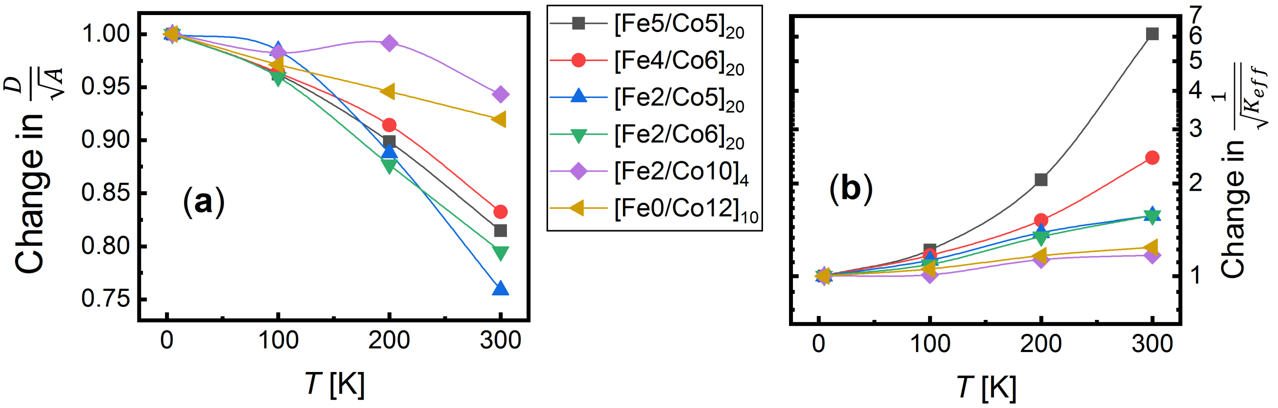

Figure S14: Variation of **(a)** $\frac{D}{\sqrt{A}}$ and **(b)** $\frac{1}{\sqrt{K_{eff}}}$ across various samples with T.

3 Temperature dependent $\rho^{THE}(H)$ profiles

Figure S15 shows the T-dependent $\rho^{THE}(H)$ profiles for samples presented in figure 2a of the main paper. Please note that the profiles show a small non-zero offset above the saturation field of magnetization ($H > H_s$), as explained in the methods section of the paper. This is a systematic offset due to the fitting procedure. The THE does not change sign.

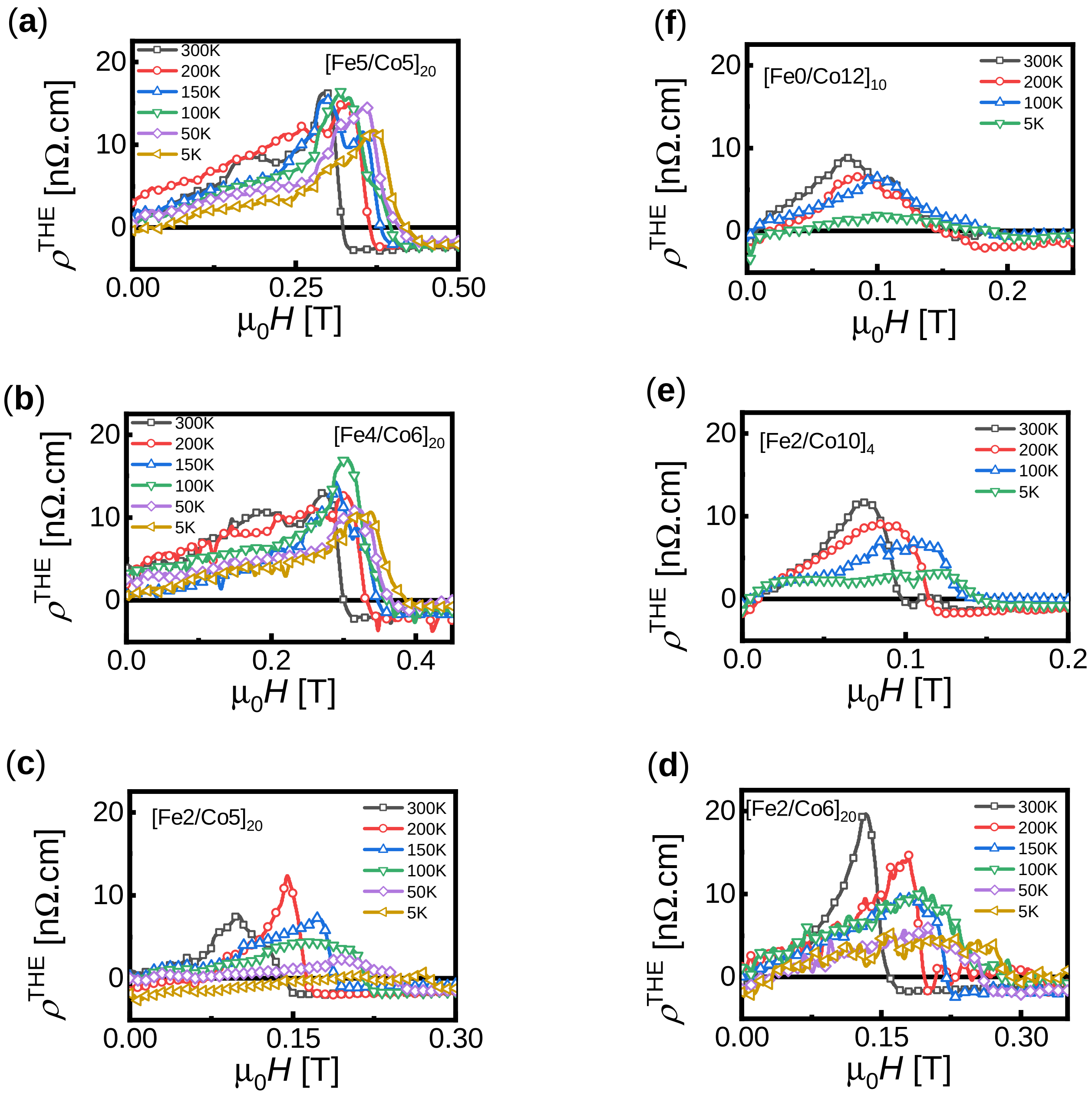

Figure S15: Temperature dependent $\rho^{THE}(H)$ profiles with varying Fe/Co composition and stack repeats presented in Fig. 2a-c of the main paper.

3.1 $\rho^{THE}(H)$ profiles with Fe/Co composition and the number of repeats in a stack

Figure S16 shows the variation of $\rho^{THE}(H)$ with temperature for varying numbers of Fe/Co layer repeats in a multilayer stack. These trends confirm that in our multilayers, the magnetic interactions leading to the stabilization of skyrmionic textures (and hence ρ^{THE}) are sensitive to the Fe/Co composition, number of Fe/Co repeats in a stack and temperature. While the Fe/Co ratio predominantly tunes the Dzyaloshinskii-Moriya interaction (DMI), the dipolar interaction between the magnetic layers becomes increasingly important as the number of [Fe/Co] layer repetitions increases. This dipolar interaction also helps to stabilise relatively larger-sized skyrmions compared to DMI. For the temperature range ($5-300$ K) used in our experiments, by adjusting the number of repeats in a Co-rich interface (in this case Fe2/Co10), one can access zones of the phase diagram covering isolated skyrmions (larger ρ^{THE}) and the saturated ferromagnetic phase ($\rho^{THE} \approx 0$). On the other hand, using Fe-rich interfaces (in this case Fe5/Co5), one can also stabilize a disordered skyrmion lattice (relatively smaller ρ^{THE})

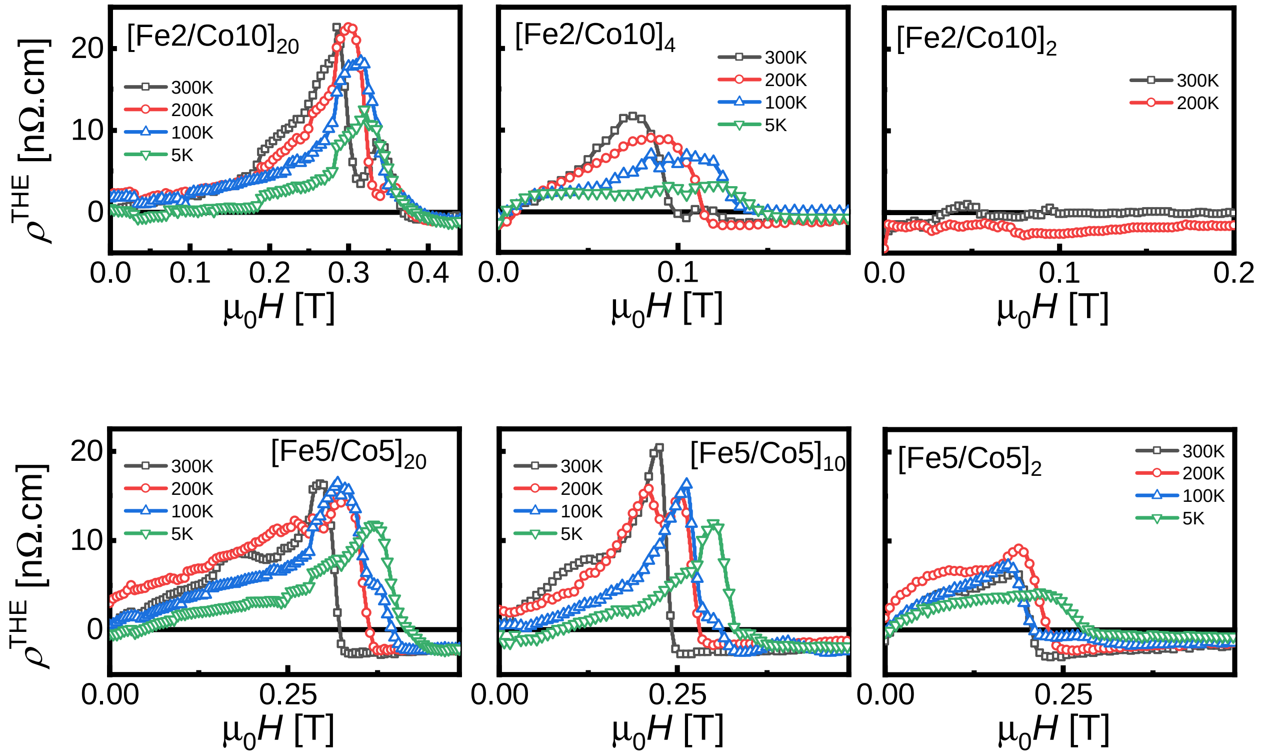

Figure S16: **Evolution of $\rho^{THE}(H)$ with varying numbers of Fe/Co layer repeats in a multilayer stack for two fixed Fe/Co compositions:** Fe2/Co10 (top row) and Fe5/Co5 (bottom row).

4 Correlation between the enhancement in ρ^{THE}, skyrmion configuration transition and sign reversal of R_0

In Fig. S17 we show how the local enhancement in ρ^{THE} discussed in figure 2b-c of the main paper correlates with the sign reversal of R_0. We find that the local enhancement in ρ^{THE} always occurs at a temperature higher than or equal to the sign reversal of R_0 (S17a-c). Similar behaviour is observed upon varying κ (via the Fe/Co composition) at fixed temperature (S17d-e), with the ρ^{THE} peak occurring at higher κ and R_0 values as large as -3 to -11 nΩ.cm/T, which are considerably larger than the tiny values recorded close to the sign reversal. This systematic behaviour in ρ^{THE} and sign reversal in R_0 can also be correlated with the transition in skyrmion configuration from an isolated to a lattice phase. The close connection between electrical transport and emerging magnetic textures suggests that both these factors are influenced by systematic variations in the occupancy of the electronic bands while varying the temperature and composition of our films. It is tremendously difficult to extract quantitative information from the band structure of these multilayer stacks, due to the presence of large number of bands. However, in the following sections we use a tight binding model and DFT calculations to qualitatively explain the sign reversal of R_0 without affecting the sign of ρ^{THE}, as well as the systematic band shift with respect to the Fermi energy upon changing the Fe/Co composition.

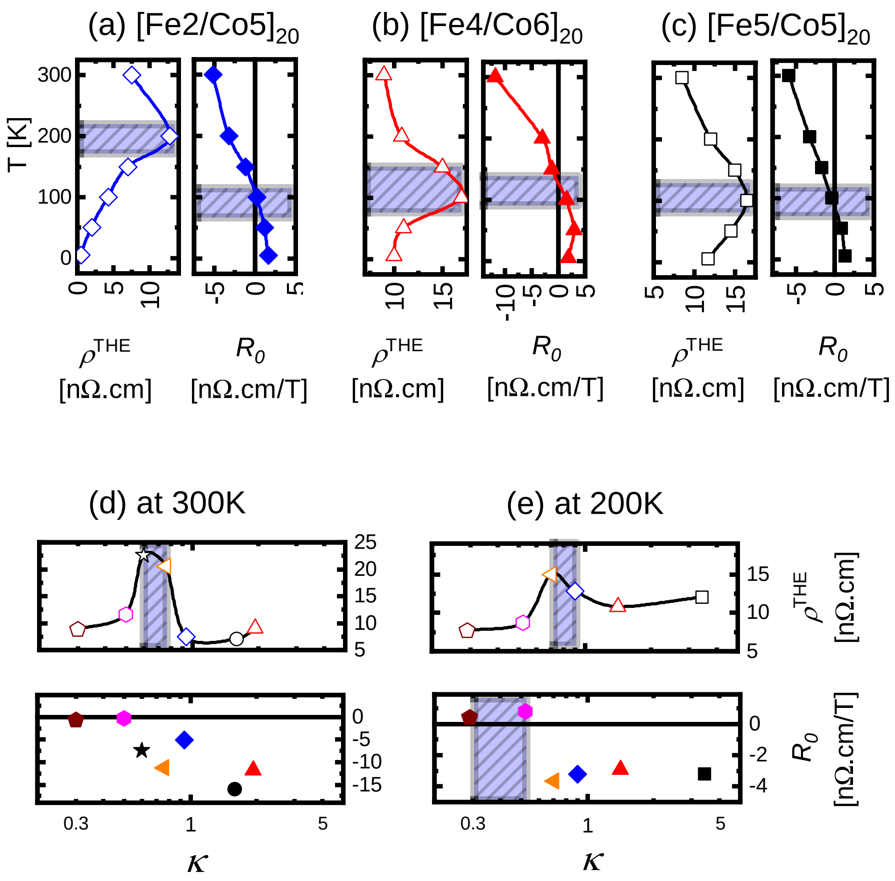

Figure S17: **Evolution of the local maximum in ρ^{THE} and the sign reversal of R_0** with temperature for fixed Fe/Co compositions **(a-c)** and varying κ (through Fe/Co compositions) at fixed temperatures **(d-e)**. Shaded regions in each panel mark the temperatures at which ρ^{THE} shows a local maximum and R_0 undergoes a sign reversal. Note that the sign of R_0 remains the same at 300 K and the peak of ρ^{THE} occurs between -7 to -11 nΩ.cm/T. The plots of ρ^{THE} are reproduced from figure 2 of the main paper.

5 Insights into the Hall effect in a multi-band system

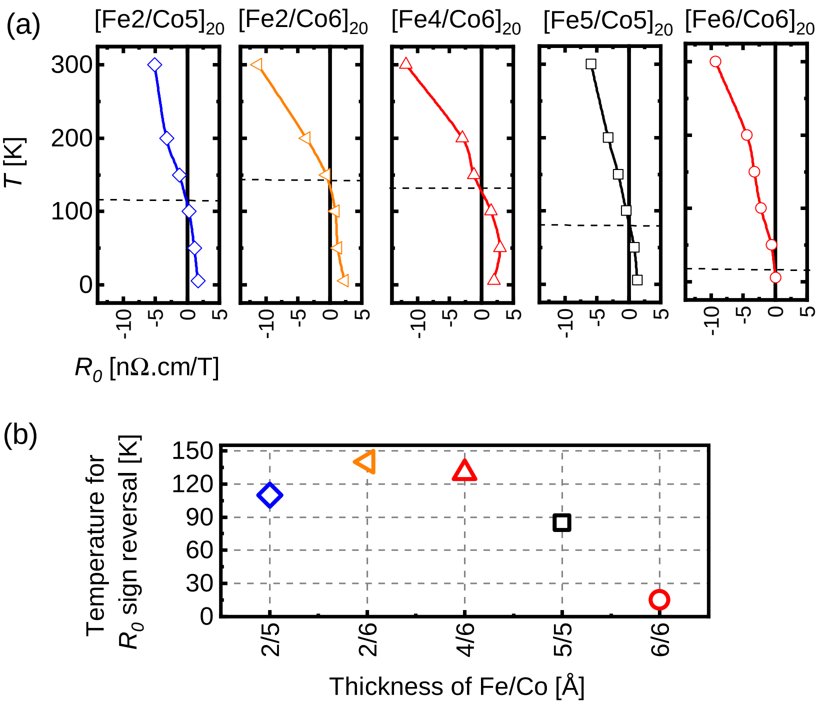

Figure S18: **Role of temperature and Fe/Co layer thickness on the classical Hall coefficient** (R_0)**: (a)** R_0 evolution with T for varying Fe/Co layer thicknesses, dotted lines in panel (a) mark the sign reversal of R_0. **(b)** Effect of Fe/Co layer thicknesses on R_0 sign reversal.

As shown in Fig. S18, our T-dependent Hall transport suggests a sign change of the classical Hall coefficient (R_0) when T is increased, and it is sensitive to the relative thickness of the Fe/Co layers. However, we find a topological Hall effect (THE) that still has the same sign (see above section and Fig. S15). This will be explained in the following.

We have conducted *ab-initio* calculations of the measured systems (shown in the next section). However, the band structure of a multi-stack is highly complicated. Even for a single stack of Pt(5)/Co(3)/Fe(2)/Ir(5) the unit cell consists of 30 atoms, leading to many overlapping bands near the Fermi energy. This renders a direct calculation of the Hall resistivity impossible. However, we can extract the fundamental features of the carriers from the band structure to qualitatively understand the origin of the unconventional sign reversal behavior in R_0 and THE.

5.1 Semiclassical argumentation

The existence of a large number of bands provides a variety of effective masses of the carriers. In particular, we find electron and hole states with various magnitudes of effective masses near the Fermi energy. This was a motivation to construct a two-orbital tight-binding model that allows to explain the experimental findings qualitatively. We start our explanation with semi-classical considerations for the transport in semiconductors.

We construct the model such that near the Fermi energy $E_F = 0$, electron states and hole states with different effective masses are present (Fig. S19a). The spins in these two bands are oriented parallel ($\uparrow\uparrow$) and anti-parallel ($\uparrow\downarrow$) with respect to the texture, respectively. This is accounted for in the tight-binding matrix via a Hund's coupling term by analogy with Ref. [7] (see the following section for more details). As the spin texture we consider a skyrmionic phase for the THE and a collinear ferromagnet for the classical Hall effect.

Typically, the Hall resistivity is given by

$$\rho_{xy} = \frac{p\mu_h^2}{e(p\mu_h + n\mu_e)^2} B_h - \frac{n\mu_e^2}{e(p\mu_h + n\mu_e)^2} B_e \tag{2}$$

when electrons (carrier density n and mobility μ_e) and holes (carrier density p and mobility μ_h) are present. In the presence of an external magnetic field and a ferromagnetic spin texture, as in the ordinary Hall effect scenario, the effective magnetic field is given exclusively by the actual field. Both carrier species feel the same magnetic field $B_h = B_e = B$. For this reason, the sign of the resistivity is determined by the sign of $p\mu_h^2 - n\mu_e^2$. Under variation of the T, the dominating carrier density can change from hole-like to electron-like. For two bands, this is only possible if both carriers have different effective masses. Simply speaking, for elevated T, more higher energetic states (i. e. electronic states) are occupied than lower energetic states (i. e. hole states).

Concerning the THE, the emergent field of the skyrmion acts differently on the two different types of carriers that are characterized by spin parallel and spin anti-parallel states, respectively, in our model. In the adiabatic limit, the electron spins align with the spin texture upon traversing a skyrmion. Therefore, the different spin species align oppositely and feel opposite emergent fields. Considering only the skyrmion texture and no external field, the effective magnetic fields are $B_e = -B_h = B_{\mathrm{em}}$. For this reason, the sign of the Hall resistivity is given by $-(p\mu_h^2 + n\mu_e^2)B_{\mathrm{em}}$ which does not change its sign under variation of the two carrier densities.

5.2 Explicit transport calculation using Berry curvature

Next, we show explicit calculations using the four-band tight-binding model to add credence to the above interpretation. The model has been constructed such that it resembles a system which exhibits a sign change in the classical Hall conductivity upon variation of the T. At the same time, the sign of the topological Hall conductivity remains, like in our experimental observations in metallic multilayers.

The model Hamiltonian features nearest-neighbor hopping (amplitudes t) of electrons and a coupling to the texture via Hund's coupling (amplitude m, vector of Pauli matrices $\boldsymbol{\sigma}$)

$$H = \sum_{i,\alpha} o_\alpha c_{i,\alpha}^\dagger c_{i,\alpha} + \sum_{\langle i,j\rangle,\alpha,\beta} t_{ij}^{(\alpha\beta)} c_{i\alpha}^\dagger c_{j\beta} + m \sum_{i,\alpha} \boldsymbol{m}_i \cdot (c_{i\alpha}^\dagger \boldsymbol{\sigma} c_{i\alpha}), \tag{3}$$

where $c_i^\dagger$, c_i are creation and annihilation operators at site i with orbital α. In all calculated systems, we consider two orbitals $\{\alpha, \beta\} = \{1, 2\}$ which – for simplicity – are assumed to not hybridize: $|t_{ij}^{(\alpha,\beta)}| = t_\alpha \delta_{\alpha\beta}$. Here, $\delta_{\alpha\beta}$ is the Kronecker delta. The nearest-neighbor hopping amplitudes for the two orbitals have different magnitudes: $t_1 = -1\,\mathrm{eV}$ and $t_2 = 0.5\,\mathrm{eV}$. The coupling to the texture is $m = 5\,\mathrm{eV}$, and the on-site energies for the two orbitals are $o_1 = -1\,\mathrm{eV}$ and $o_2 = 4\,\mathrm{eV}$.

In the following, the classical and the topological Hall scenarios will be discussed. In both cases, the tight-binding Hamiltonian is diagonalized where the eigenvalues are the band energies $E_n(\boldsymbol{k})$ and the eigenvectors $|n(\boldsymbol{k})\rangle$ are important for the calculation of the reciprocal-space Berry curvature

$$\Omega_n^z(\boldsymbol{k}) = -2\mathrm{Im} \sum_{m\neq n} \frac{\langle n(\boldsymbol{k})|\partial_{k_x} H(\boldsymbol{k})|m(\boldsymbol{k})\rangle \langle m(\boldsymbol{k})|\partial_{k_y} H(\boldsymbol{k})|n(\boldsymbol{k})\rangle}{(E_n(\boldsymbol{k}) - E_m(\boldsymbol{k}))^2}. \tag{4}$$

This quantity determines the intrinsic contribution to the Hall conductivity

$$\sigma_{xy}(E_F) = -\frac{e^2}{h}\frac{1}{2\pi} \sum_n \int \Omega_n^z(\boldsymbol{k}) f[E_n(\boldsymbol{k}) - E_F]\, \mathrm{d}^2 k. \tag{5}$$

f is the Fermi distribution function that accounts for the reoccupation of the electronic states upon variation of the temperature

$$f(E) = [\exp(E/(k_B T)) + 1]^{-1}. \tag{6}$$

Ordinary Hall conductivity. First, we discuss the results for a ferromagnetic texture in the presence of an external magnetic field. This scenario brings about an ordinary Hall effect. In this case, the texture is $\boldsymbol{m}_i \equiv \boldsymbol{e}_z$. Without the presence of an external magnetic field, the unit cell of the considered square lattice comprises a single lattice site with two orbitals. The resulting band structure exhibits four bands as shown in Fig. S19a.

The presence of an external magnetic field modifies the hoppings

$$t_{ij}^{(\alpha\alpha)} = t_\alpha \exp\left(i\frac{e}{\hbar} \int_{\boldsymbol{r}_i \to \boldsymbol{r}_j} \boldsymbol{A}(\boldsymbol{r}) \cdot \mathrm{d}\boldsymbol{l} \right). \tag{7}$$

Here, the vector potential $\boldsymbol{A}$ can be expressed in Landau gauge to fulfill $\boldsymbol{B} = \nabla \times \boldsymbol{A}$. As a detail, it is worth mentioning that the Peierls phase in the hopping has to be periodic. For certain magnetic field strengths

$$B = \frac{p}{q}\frac{h}{e}\frac{1}{a^2} \tag{8}$$

a magnetic unit cell can be constructed. Here, p and q have to be coprime integers. The smaller q, the smaller the unit cell and the faster the calculation. For this reason, we chose $p/q = 1/36$ which would correspond to a large field. This is unproblematic, since the strength of the magnetic field does not affect the energy and T dependence of the Hall conductivity qualitatively but only scales its magnitude.

The resulting band structure consists of many Landau levels (cf. Ref. [7] for more details). The energy dependent Hall conductivity exhibits quantized values in multiples of e^2/h in the band gaps at zero T. For finite T, these plateaus are smeared out resulting in smooth curves as shown in Fig. S19c.

The sign of the conductivity is given by the dominant electronic character and the sign of the external field as can intuitively be understood by the Lorentz force $m\ddot{\boldsymbol{r}} = q\dot{\boldsymbol{r}} \times \boldsymbol{B}$. For small T, the predominant carrier type can be deduced directly from the zero-field band structure. For example, near the bottom of the lowest band in Fig. S19a, the band structure resembles a free electron parabola, which is why the Hall conductivity is negative at these energies. Near the Fermi energy, electron and hole states are present. Since these states have different effective masses, an increase in the T leads to a change of the predominant carrier type, which leads to a change in the sign of the Hall conductivity. In Fig. S19d the T dependence is shown with a sign change at a critical T.

Topological Hall conductivity. Secondly, we discuss the topological Hall effect of electrons in skyrmion lattice without taking into account the magnetic field, in order to calculate the isolated topological contribution. This time the magnetic texture is space dependent. The texture in the magnetic unit cell is given as

$$\boldsymbol{m}_i(\boldsymbol{r}_i) = \begin{pmatrix} \sin(\frac{\pi}{r_0} r_i)\frac{x_i}{r_i} \\ \sin(\frac{\pi}{r_0} r_i)\frac{y}{r_i} \\ -\cos(\frac{\pi}{r_0} r_i) \end{pmatrix} \tag{9}$$

for $r_i < r_0$, where $r_i = \sqrt{x_i^2 + y_i^2}$ is measured with respect to the skyrmion's center. This magnetization resembles circular Néel skyrmions (topological charge $N_{\mathrm{Sk}} = -1$) in a square lattice. For the calculations we assume a skyrmion radius of $r_0 = 5a$ (here a is the lattice constant) and a square shaped unit cell comprising 100 lattice sites.

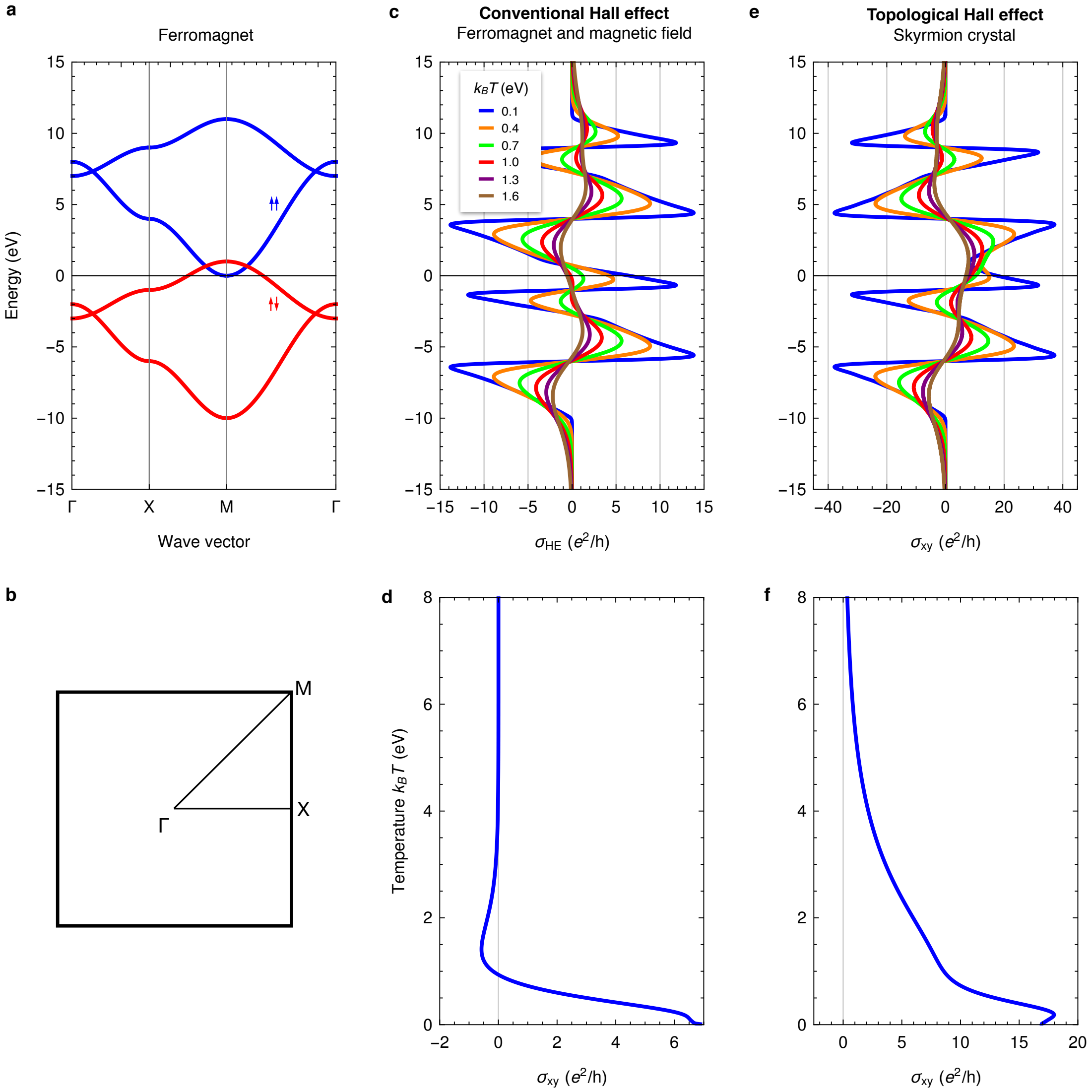

Figure S19: **Ordinary and topological Hall effects in a tight-binding model.** (a) The zero-field band structure in the presence of a ferromagnetic texture. Blue and red indicate parallel and anti-parallel spin alignment, respectively. The Brillouin zone of the square lattice is shown in (b). In (c) the energy dependence of the Hall conductivity is shown in the presence of an external magnetic field and a collinear texture for different temperatures. The Fermi energy is $E_F = 0$. (d) shows the temperature dependence of this Hall conductivity with a sign change occurring at a critical temperature. Panels (e) and (f) show the topological Hall conductivity in the presence of a skyrmion crystal without external magnetic field.

Upon traversing the skyrmionic texture, the conduction electrons reorient their spin (partially) parallel or anti-parallel with respect to the texture. Due to the opposite spin alignment for the two bands near the Fermi energy, the emergent fields have opposite signs for the two spin species implying fundamentally different transport properties compared to the classical Hall effect. This time, the sign of the Hall conductivity (cf. Fig. S19e for different T) is not solely given by the effective mass but also by the spin species. For this reason, the electron-like spin up states and the hole-like spin down states near the Fermi energy give rise to the same sign of the Hall conductivity which is why no sign change occurs upon variation of the T (Fig. S19f), different to the behavior of the ordinary Hall effect (Fig. S19d), as discussed above.

6 Density functional theory calculations

In order to corroborate the tight-binding model discussed above, we performed ab-initio calculations of different stacks. We used the QuantumATK package [8] together with the GGA-PBE exchange-correlation functional [9] and the SG15 combination of norm-conserving pseudopotentials and LCAO basis sets [10]. We used a dense $24 \times 24 \times 1$ $\mathbf{k}$-point grid and a density mesh cutoff of 160 Hartree. The total energy and forces have been converged at least to 10^{-4} eV and 0.01 eV/Å, respectively. Spin-orbit coupling is included in band structure calculations. The minimal setup is a single stack consisting of Pt(10)/Co(6)/Fe(4)/Ir(10). Additionally, we add another Pt(10) since in the experiment no free Ir surface is present. We calculate a collinear ferromagnet; the self-consistent calculation of a skyrmion texture is too demanding by todays standards. Still, the unit cell consists of many atoms leading to many bands in the band structure (Fig. S20). This renders a direct calculation of the Hall conductivity impossible.

In Fig. S20, the bands have been colored blue if the Co character is dominant and red if Fe is dominant. The bands also hybridize with Ir and Pt to a large degree but we decided to focus here on the magnetic elements. Near the K point the motive of the tight-binding model becomes visible. We observe electron and hole bands with different effective masses and different magnetic compositions. This explains that the peculiar sign change of the Hall coefficient can indeed occur upon variation of the T. Of course, here the situation is more complex than in the four-band tight-binding model but the general arguments remain.

Next, we will show how the motive in the band structure changes upon changing the stack. In the experiment, the multistack consists of several repetitions. In the calculations, we can only calculate up to three repetitions in a reasonable time. As is shown in Fig. S21, a repetition of the stack adds more bands to the band structure. However, the copies look

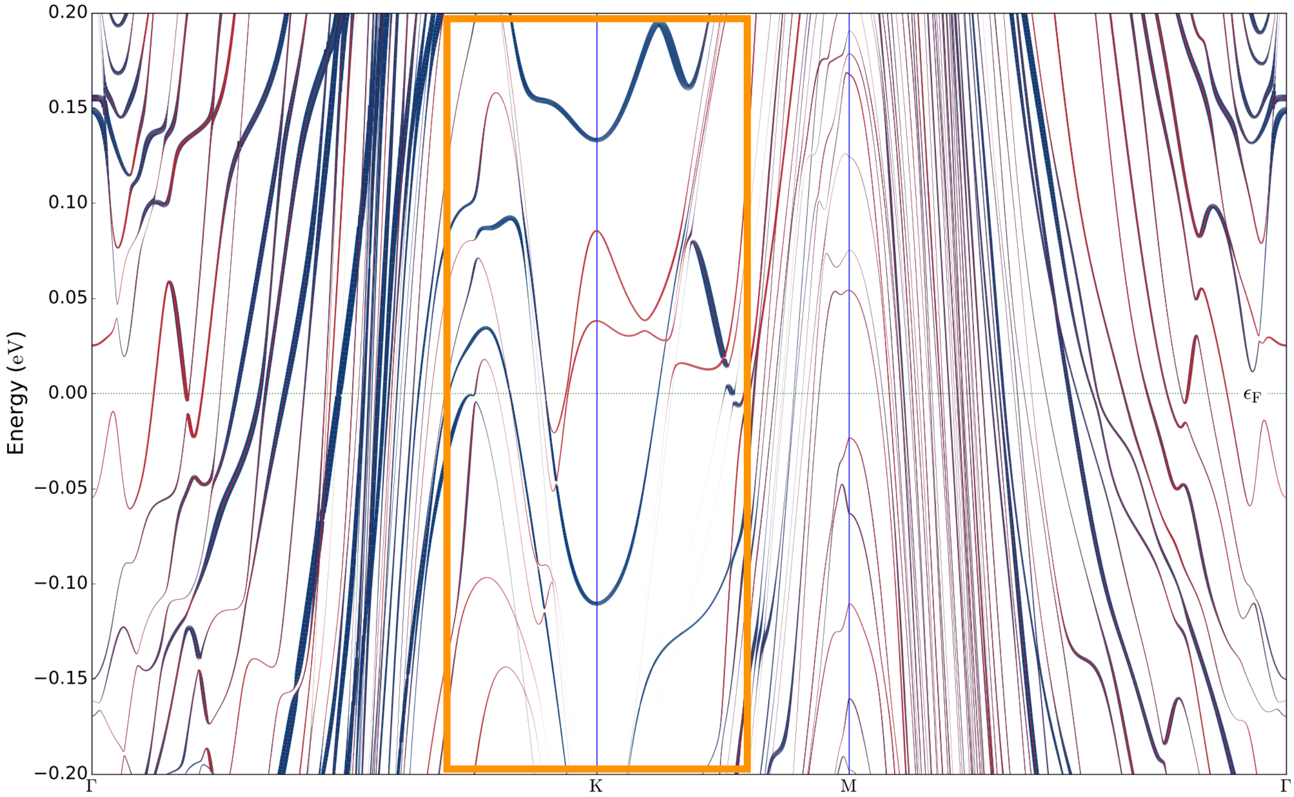

Figure S20: **Ab-initio band structure of a single stack Pt(10)/Co(6)/Fe(4)/Ir(10)/Pt(10)**. Blue bands are dominated by Co and red bands are dominated by Fe. The highlighted region exhibits electron and hole bands with different masses. The evolution of these bands under variation of the stack will be investigated in Figs. S21 and S22.

rather similar to the initial bands implying that the repetition rate plays a minor role for the Hall conductivity.

This changes when the composition of the stack is changed. Exemplarily, we vary the Fe thickness (Fig. S22), where panel b is the highlighted region of Fig. S20. Compared to Fe(4), a decrease of the Fe thickness leads to a shift of the Fe bands in the negative energy direction (Fig. S22a). Reversely, an increase in the thickness shifts the Fe bands slightly upwards in energy (Fig. S22c).

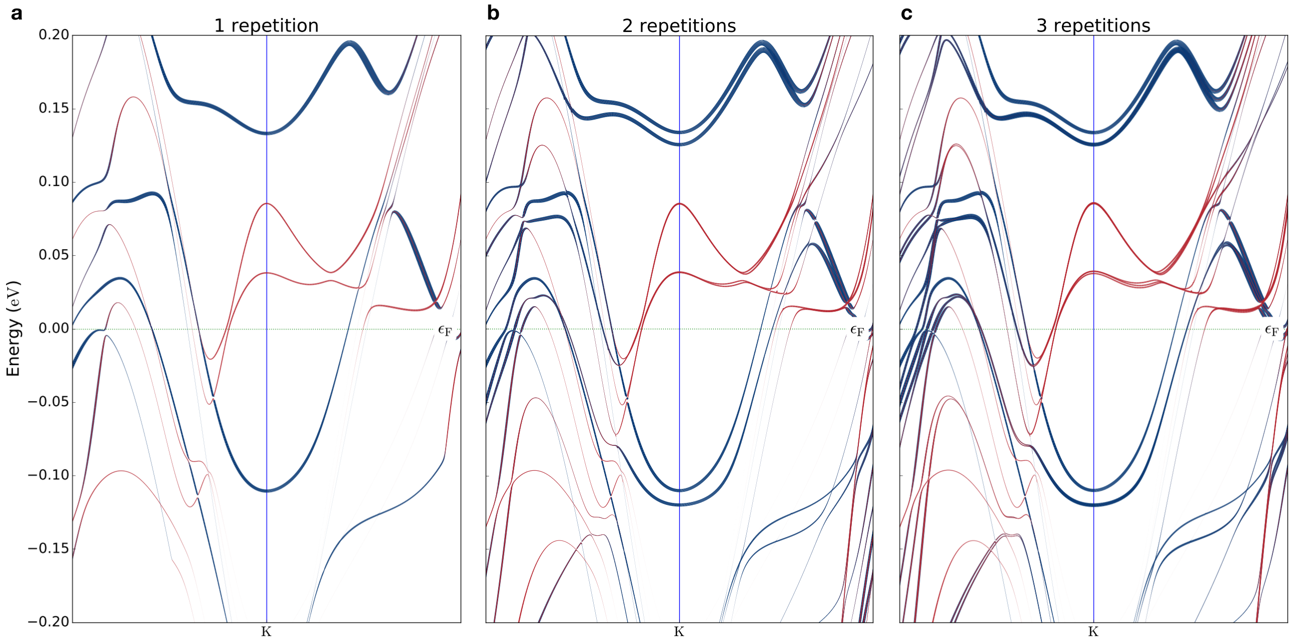

Figure S21: **Influence of the repetition rate.** Similar to the orange region of Fig. S20 but the repetition number of [Pt(10)/Co(6)/Fe(4)/Ir(10)] is changed as indicated.

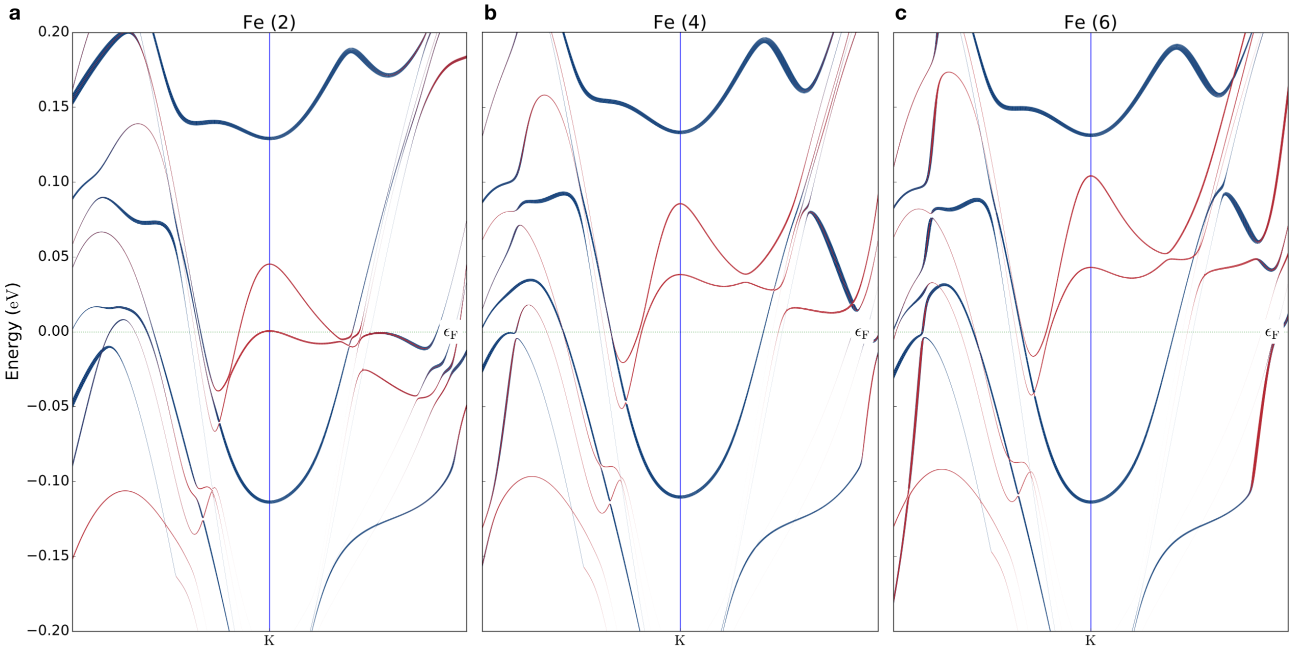

Figure S22: **Influence of the composition.** Similar to the orange region of Fig. S20 but the Fe composition in [Pt(10)/Co(6)/Fe(x)/Ir(10)] is changed as indicated.

References

[1] Moreau-Luchaire, C. et al. Additive interfacial chiral interaction in multilayers for stabilization of small individual skyrmions at room temperature. Nat. Nanotechnol. **11**, 444–448 (2016).

[2] Woo, S. et al. Observation of room-temperature magnetic skyrmions and their current-driven dynamics in ultrathin metallic ferromagnets. Nat. Mater. **15**, 501–506 (2016).

[3] Soumyanarayanan, A. et al. Tunable room-temperature magnetic skyrmions in Ir/Fe/Co/Pt multilayers. Nat. Mater. **16**, 898 (2017).

[4] Yagil, A. et al. Stray field signatures of Néel textured skyrmions in Ir/Fe/Co/Pt multilayer films. Appl. Phys. Lett. **112**, 192403 (2018).

[5] Raju, M. et al. The evolution of skyrmions in Ir/Fe/Co/Pt multilayers and their topological Hall signature. Nat. Commun. **10**, 696 (2019).

[6] Tomasello, R. et al. Origin of temperature and field dependence of magnetic skyrmion size in ultrathin nanodots. Phys. Rev. B. **97**, 060402 (2018).

[7] Göbel, B., Mook, A., Henk, J. & Mertig, I. Unconventional topological Hall effect in skyrmion crystals caused by the topology of the lattice. Phys. Rev. B. **95**, 094413 (2017).

[8] Smidstrup, S. et al. Quantumatk: An integrated platform of electronic and atomic-scale modelling tools. Journal of Physics: Condensed Matter **32**, 015901 (2019).

[9] Perdew, J. P., Burke, K. & Ernzerhof, M. Generalized gradient approximation made simple. Phys. Rev. Lett. **77**, 3865 (1996).

[10] Schlipf, M. & Gygi, F. Optimization algorithm for the generation of oncv pseudopotentials. Computer Physics Communications **196**, 36–44 (2015).
